# Microbiomics: The Next Pillar of Precision Medicine and Its Role in African Healthcare

**DOI:** 10.3389/fgene.2022.869610

**Published:** 2022-04-05

**Authors:** C. K. Nkera-Gutabara, R. Kerr, J. Scholefield, S. Hazelhurst, J. Naidoo

**Affiliations:** ^1^ Sydney Brenner Institute for Molecular Bioscience (SBIMB), Faculty of Health Sciences, University of the Witwatersrand, Johannesburg, South Africa; ^2^ Bioengineering and Integrated Genomics Research Group, Council for Scientific and Industrial Research (CSIR), Pretoria, South Africa; ^3^ Division of Human Genetics, National Health Laboratory Service and School of Pathology, Faculty of Health Sciences, University of the Witwatersrand, Johannesburg, South Africa; ^4^ School of Electrical and Information Engineering, University of the Witwatersrand, Johannesburg, South Africa

**Keywords:** microbiome, precision medicine, gut health and disease, African health care, microbiome mapping

## Abstract

Limited access to technologies that support early monitoring of disease risk and a poor understanding of the geographically unique biological and environmental factors underlying disease, represent significant barriers to improved health outcomes and precision medicine efforts in low to middle income countries. These challenges are further compounded by the rich genetic diversity harboured within Southern Africa thus necessitating alternative strategies for the prediction of disease risk and clinical outcomes in regions where accessibility to personalized healthcare remains limited. The human microbiome refers to the community of microorganisms (bacteria, archaea, fungi and viruses) that co-inhabit the human body. Perturbation of the natural balance of the gut microbiome has been associated with a number of human pathologies, and the microbiome has recently emerged as a critical determinant of drug pharmacokinetics and immunomodulation. The human microbiome should therefore not be omitted from any comprehensive effort towards stratified healthcare and would provide an invaluable and orthogonal approach to existing precision medicine strategies. Recent studies have highlighted the overarching effect of geography on gut microbial diversity as it relates to human health. Health insights from international microbiome datasets are however not yet verified in context of the vast geographical diversity that exists throughout the African continent. In this commentary we discuss microbiome research in Africa and its role in future precision medicine initiatives across the African continent.

## The Human Microbiome as a Focal Point for Precision Medicine in Africa

Genomic medicine catalysed a change in ideology around the significance of genetic diversity in the provision of healthcare, giving rise to precision medicine, which guides pharmaceutical intervention to improve clinical outcomes (McCarthy et al., 2013; Schork, 2015; Kuntz and Gilbert, 2017). While human genomics continues to remain central to most current precision strategies, there is also an increasing appreciation for the value of integrated multi-omics approaches in better understanding clinical diversity and developing a more comprehensive outlook towards precision medicine (Olivier et al., 2019).

The human microbiome refers to the collection of symbiotic, pathogenic and commensal microorganisms (e.g., bacteria, archaea, viruses and fungi) that cohabit discrete anatomical sites across the human body (Lederberg, 2000). The total number of human cells in the average body is surpassed by the number of microbes it houses, while the unique genetic information encoded by these microbial communities (∼3 million genes) also dwarf that of the human genome (∼23,000 genes) (Qin et al., 2010; Sender et al., 2016). Increasing accessibility to next generation sequencing (NGS) technologies have catalysed advances in microbiome research and illuminated its role in human health, prompting its moniker as our “second genome” (Grice and Segre, 2012; Cani, 2018; Tierney et al., 2021).

While the general composition of the gut microbiome remains quasi-stable and unique for an individual throughout adult life, it is also heavily shaped by factors like diet, lifestyle practices and environmental exposures (Tidjani Alou et al., 2016; Vangay et al., 2018). Notably, environmental factors associated with diet and lifestyle account for ∼20% of gut microbiome variance where genetically unrelated individuals residing in close proximity are more likely to exhibit similar microbiome profiles than genetically related individuals who did not (Rothschild et al., 2018). In fact less than 2% of microbiome diversity can be explained by host genetics thus further strengthening the argument that the human microbiome may serve as a valuable complementary approach to traditional genomic medicine (Rothschild et al., 2018).

With continued industrialisation of human habitats and the widespread adoption of westernised lifestyles and diets, the human microbiome is being reshaped to reflect these changes (Rothschild et al., 2018; Vallès and Francino, 2018; Vangay et al., 2018). Over the last two decades African populations have experienced rapid transitions to an increasingly westernized nutrition and lifestyle, associated with an increased prevalence of non-communicable diseases (NCDs) like obesity, cancer and various neurological disorders (Abrahams et al., 2011). South Africa was recently ranked as the unhealthiest country in the world by the Indigo Wellness Index which is based on metrics including healthy life expectancy, obesity, depression and government expenditure on healthcare (Indigo Wellness Index, 2019). The added burden of infectious disease in addition to NCDs, and limited resources therefore necessitate innovative strategies to democratise access to precision healthcare on the continent.

Due to the dynamic nature of the microbiome, the timescale at which it reacts to environmental and health status changes is significantly shorter than the onset of the associated effects on health (Vallès and Francino, 2018; Vangay et al., 2018). Importantly microbiome profiling of an individual’s gut is also significantly more cost effective (∼10x) than sequencing an individual’s genome. Unlike our base genetics, our “second genome” can also be readily and specifically modulated by a number of non-invasive, acute interventions like the use of probiotics, prebiotics, dietary inputs, lifestyle changes etc. (Dethlefsen and Relman, 2011; Maurice et al., 2013). This malleable property of the microbiome together with its strong valuable proposition as a biomarker, position it as an attractive module for precision medicine strategies (Tidjani Alou et al., 2016; Thursby and Juge, 2017). This is of particular relevance in the African continent where the use of the microbiome as both 1) a cost effective biomarker with the ability to predict early risk for multiple diseases and 2) a readily amenable target for non-invasive and cost effective therapeutic intervention (e.g probiotics) could prove highly impactful. In this commentary we discuss the human gut microbiome, its relevance to healthcare challenges faced by the African continent and the requirement for a coordinated and unified approach to large-scale microbiome research efforts across Africa.

## Linking the Human Microbiome to African Health Issues

Dysbiosis, the perturbation of the natural balance of microbes within the human body, is associated with several human pathologies ranging from immune disorders such as asthma (Frati et al., 2018; Stokholm et al., 2018) and inflammatory bowel diseases (IBDs) (Xavier and Podolsky, 2007; Kostic et al., 2014) to metabolic disorders like obesity, malnutrition, diabetes and even gluten intolerance (Larsen et al., 2010; Musso et al., 2010; Nylund et al., 2014; Tidjani Alou et al., 2016).

Of particular relevance to the African continent, significant alterations to the gut microbiome have been noted in: infectious diseases like Human Immunodeficiency Virus (HIV) (reviewed by (Koay et al., 2018) and tuberculosis (TB) (reviewed by (Liu Y. et al., 2021); as well as other emerging health threats like Parkinson’s disease (Scheperjans et al., 2015; Tremlett et al., 2017; Vogt et al., 2017); mental health (Bhattacharjee and Lukiw, 2013; Foster and McVey Neufeld, 2013; Kelly et al., 2016); cardiovascular disease (Feng et al., 2016; Jie et al., 2017) and cancer (reviewed by (Lee et al., 2021). While further studies are still required to validate causality versus consequence with regards to dysbiosis and certain diseases, there is clear potential for the microbiome as either a biomarker, therapeutic target or both for some of the major health challenges faced by the African continent, as reviewed below.

### Obesity

The prevalence of obesity in African populations doubled between 1990 and 2016, to over 10 million individuals (World Health Organization, 2016; Biadgilign et al., 2017). Obesity has been directly linked to various NCDs including type II diabetes which has increased by over 120% in Africa since 1980, with a predicted economic consequence of $60 billion United State Dollars (USD) by 2030 in sub-Saharan Africa alone (Jaffar and Gill, 2017). Clinical and experimental studies in human cohorts have established correlations between gut microbiota and obesity, through baseline microbial composition having a direct impact on improvement in insulin sensitivity and halting progression of type I diabetes (Kootte et al., 2017; de Groot et al., 2021). Longitudinal studies in the United Kingdom (United Kingdom) and the United States have also shown that there is an increased risk of neurocognitive deficits and autism spectrum disorders (ASDs) associated with maternal obesity (Basatemur et al., 2013; Pugh et al., 2015). Murine models revealed that maternal high-fat diets (mHFD) induced long-term cognitive deficits that span across several generations (Sarker and Peleg-Raibstein, 2018). This was further confirmed by recent evidence that maternal obesity disrupted the gut microbiome in offspring and that this was strongly associated with cognitive and social dysfunctions (Liu X. et al., 2021). Given the significant prevalence of obesity (>20%) of women above 20 years of age in South Africa (Lobstein and Brinsden, 2017), obesity could pose a serious long term challenge to health on the African continent.

Microbiome-linked therapies in response to maternal obesity-related deficits have provided promising interventions using high-fibre diet supplementation to produce microbiota-derived short-chain fatty acids that alleviate behavioural deficits via the gut-brain axis (Liu X. et al., 2021). Additionally mouse models of diet-induced obesity have demonstrated the bacterium, *A. muciniphila*’s, inverse correlation with obesity, where its presence correlated with the restoration of damaged gut structure, improved hepatic function and glucose homeostasis in high fat-diet-fed mice (Everard et al., 2013; de Vos, 2017; Yang et al., 2020). The value of early interventions against childhood immune disorders have also been successfully demonstrated with *Bifidobacterium infantis*, whereby stable and persistent colonization and remodelling of the infant intestinal microbiome was accomplished, resulting in reduced enteric inflammation (Grześkowiak et al., 2012; Huda et al., 2014; Frese et al., 2017; Henrick et al., 2019). The innovative utilisation of microbiome modulation strategies, like faecal microbiota transplantation and probiotic intervention along with microbiome profiling have great potential for the early diagnosis and treatment of metabolic disorders like obesity, prior to the onset of disease (Khanna et al., 2017; Depommier et al., 2019; Lam et al., 2019; Sorbara and Pamer, 2022).

### Neurodegenerative Disorders and the Gut-Brain Axis

African populations have experienced an increase in life expectancy, and with an aging population comes the increased burden of age-related disorders (Dekker et al., 2020). Aging is accompanied by chronic inflammation, increased intestinal permeability, disrupted nutrient absorption and impaired digestion, all exhibiting bidirectional interactions with the gut microbiome, termed the gut-brain axis (An et al., 2018).

Studies of neurodegenerative diseases (ND) such as amyotrophic lateral sclerosis (ALS) and Parkinson’s disease have revealed distinct disease associated gut microbial signatures in cohort studies along with evidence of microbial protection against disease progression caused by antibiotic-induced dysbiosis (Petrov et al., 2017; Peng et al., 2018; Blacher et al., 2019; Fang et al., 2020). Animal models have shown that microbiota transplantation and probiotic treatment have the potential to ameliorate ND-related pathology and behaviour (Sun et al., 2018, 2019; Kim et al., 2020). At least 1 in 6 people suffer from anxiety and depression in South Africa, affecting a staggering 41% of pregnant women—more than three times higher than in developing countries (SADAG, 2019). Murine model research has demonstrated that decreased gut microbiota richness and diversity is associated with depression and may play a causal role in its development, further highlighting the potential of the microbiome as a therapeutic target (Kelly et al., 2016).

### Human Microbiome and SARS-CoV-2

One of the greatest global health challenges in recent times was driven by the coronavirus disease 2019 (COVID-19) pandemic. The severity of COVID-19 infection as well as the longevity of symptoms, termed “post-acute COVID-19 syndrome” (PACS), has been suggested to be heavily influenced by an individual’s gut microbiome profile (Liu et al., 2021a). Bacteria such as *Firmicutes* play a role in the enhanced expression of angiotensin-converting enzyme 2 (ACE2) in the gut, which acts as a receptor for viral entry and cell infection (Zuo et al., 2020). The findings show that at admission to hospital, distinct gut microbial signatures are present within patients and are associated with PACS at 6 months post admission (Liu et al., 2021a).

The above observation was further supported by evidence that low levels of the gut bacteria *Collinsella* coincides with COVID-19 related mortality. This bacteria produces ursodeoxycholate, which is reported to have diverse beneficial effects such as the inhibition of SARS-CoV-2 binding to the ACE2 receptor, suppression of inflammatory responses, and amelioration of acute respiratory distress syndrome in COVID-19 patients (Hirayama et al., 2021). This supports the potential for precision microbiome-based profiling, as an early warning system for the detection of PACS, as well as a target for the prevention of COVID-19 infection (Liu et al., 2021a; Hirayama et al., 2021; Yeoh et al., 2021).

The antiviral properties of the microbiome have been investigated as an alternative therapeutic approach against COVID-19 (Campbell et al., 2021; Chevallier et al., 2021; Kuzmina et al., 2021). Bacterial metabolites are able to inhibit SARS-CoV-2 infection (Piscotta et al., 2021). These metabolites were shown to exhibit structural and functional similarities to synthetic drugs examined for use against the virus, most especially N6-(D2-isopentenyl) adenosine (IPA), which exhibited greater potency and inhibitory effects than interventions such as remdesivir, fluvoxamine and favipiravir (Piscotta et al., 2021). These findings further strengthen the valuable proposition for the inclusion of the microbiome in future precision medicine efforts so that we may unlock its full potential in response to threats like COVID-19.

### Pharmacomicrobiomics and Adverse Drug Reactions

Adverse drug reactions (ADRs) refer to unintended consequences that arise from therapeutic agents administered at normal dosages and can include both inefficacy and harmful side effects. ADRs represent a significant source of patient noncompliance and clinical failure, hence a reduction in ADRs is an important goal of precision medicine (Edwards and Aronson, 2000). “Pharmacomicrobiomics” has emerged as the study of drug-microbiome interactions, or how the variations in microbial profiles can affect a drugs’ pharmacokinetics, pharmacodynamics and ultimately efficacy (Rizkallah et al., 2010; ElRakaiby et al., 2014). Evidence exists for bacterial drug metabolism as a general mechanism through which the microbiome in the gut, reproductive tracts and even disease tissue alters drug response (Zimmermann et al., 2019). In turn human-targeted (non-antibiotic) drugs have antibiotic-like side effects on gut bacteria in human cohorts, where 27% of the non-antibiotics studied inhibited the growth of at least one bacterial species (Maier et al., 2018). Over 115 drug-microbiome interactions have been documented in the PharmacoMicrobiomics database (Aziz et al., 2011). Drug-microbiome interactions modulate the bioavailability of drugs, which is important to consider for the appropriate dosing in precision medicine (Kuntz and Gilbert, 2017). Maier et al. (2018) highlight how continued pharmacomicrobiomics research aids in the achievement of a drug-microbe network that could guide drug development in line with precision medicine therapies. Much like pharmacogenomics revolutionised healthcare and led to the birth of precision medicine, pharmacomicrobiomics is quickly positioning itself as another pillar of personalized treatment (Saad et al., 2012; Maurice et al., 2013; ElRakaiby et al., 2014).

## Microbiome Research Efforts Globally and Across Africa

Geography has been purported as an important determinant of microbiome composition where geography itself may represent the confluence of multiple variables like regional dietary preferences, unique environmental exposures and ethnically diverse lifestyle practices. Li and colleagues (2014) noted geospatial clustering of gut microbial signatures within 1,200 samples of European and Asian origin while He et al. (2018) found location to be the most significant driver of gut microbial diversity across more than 7,000 participants, with common ancestry and cultural practices, within a single Chinese province. These findings would suggest that the extrapolation of complex microbiome research trends, like the characterisation of healthy and diseased states, may be confounded by geographical diversity especially in low to middle income countries (LMICs) which exhibit increased diversity and unique phyla in comparison to samples from North America and Europe (Porras and Brito, 2019). A number of large-scale efforts have thus sought to characterise microbiome diversity at various regions across the globe in order to better understand microbiome-health dynamics within specific geographical constraints.

Large scale consortium studies in North America, Europe and Asia have driven advancements in microbiome research globally (Turnbaugh et al., 2007; Ehrlich, 2011; McDonald et al., 2018; Proctor et al., 2019). The Human Microbiome Project (HMP) was a pioneering initiative that launched in the United States in 2007 with around 300 participants (Turnbaugh et al., 2007). This project made significant strides in providing communities and researchers with a wealth of data, analytical and bio-specimen resources, whilst also positioning other researchers to continue work on microbiome-linked conditions: such as pregnancy and preterm birth; IBDs; and stressors affecting pre-diabetic individuals (Fettweis et al., 2019; NIH, 2019; Proctor et al., 2019).

As a result of the ground-breaking discoveries from the HMP, the value of continued explorations to uncover the full potential of the microbiome led to other large scale projects such as: the Asian Microbiome Project launched in 2009, the American Gut Project (AGP) in 2012, the British Gut Project in 2014, and the Dutch microbiome project (2016). These projects are collaborative efforts between the Earth Microbiome Project and Human Food Project, in order to discover global microbial taxonomic and functional diversity “in the wild” and across human populations (McDonald et al., 2018). The AGP initiative used a “self-selected citizen-scientist” cohort of over 10,000 participants, collecting microbial sequence data from over 15,000 stool samples. This unprecedented scale of characterizing the human microbiome is continuously revealing novel relationships between health, lifestyle and dietary factors (Nakayama et al., 2015; McDonald et al., 2018). The momentum from these large-scale global efforts could see clinical microbiome profiling become routine practice at point of care in the developed world within the next 5 years (Harrison-Dunn, 2021). However, the lack of representation of LMICs and Africa as a whole in these early large-scale efforts would suggest a longer horizon for the implementation of such practices in these regions in the absence of proactive measures to drive microbiome research in LMICs, like those recently adopted by both China and India (Aziz et al., 2018; Porras and Brito, 2019).

Allali and colleagues (2021) compiled an illuminating systematic review of published, data-driven research articles that included human microbiome samples of African origin and the use of NGS technologies. Only 168 research articles met these criteria with ∼67% of these being published from 2017 onwards. More than 60% of countries on the African continent were represented in terms of sampling where SA, Uganda and Kenya collectively accounted for more than 40% of all studies. Intriguingly ∼80% of all studies using African microbiome samples have not been led by African research institutes or African researchers. Less than 5% of studies confirmed the recruitment of urban populations while 31% of all studies focused on recruitment in rural settings. Notably less than 4% of studies included more than 500 participants with only ∼1% of studies including more than 1,000 participants (Allali et al., 2021). These findings highlight a clear need for coordinated intra-continental, African researcher-driven microbiome initiatives that focus on addressing the future health needs of the continent. Interestingly, they also reveal gaps in current African microbiome research efforts like the under-representation of well-developed African urban environments in sampling, and the current scarcity of large-scale microbiome profiling initiatives represented in publications.

A number of African-directed initiatives have recently been launched to investigate the role of the microbiome in human health and disease on the African continent. These studies are however traditionally limited in terms of either their scale or scope when compared to large scale microbiome profiling efforts like the AGP (Allali et al., 2021). The South African Microbiome Initiative in Neuroscience, based at Stellenbosch University in SA, is a pioneering African microbiome profiling initiative (saNeuroGut, 2017). The project aims to specifically investigate the gut-brain axis and thus the role of the gut microbiome in South African neurocognitive health, particularly links to post traumatic stress disorder.

The Human Heredity and Health in Africa (H3Africa) initiative was initially established to enhance the research capacity in institutions across Africa, for establishing projects which support precision medicine for Africans (H3Africa Consortium et al., 2014). Recognising the value of the interplay between microbes and human health has led to several ongoing microbiome projects facilitated by H3Africa (Mulder et al., 2016; Mulder et al.,2018). The details of ongoing and prospective microbiome projects are outlined on the H3Africa web-interface (H3Africa, 2010) and generally involve investigating the role of the microbiome within the context of specific diseases in women and children. This includes initiatives such as the African Collaborative Center for Microbiome and Genomics Research (ACCME) based in Nigeria, studying the association between human papillomavirus (HPV) infection and the vaginal microbiome in HIV-negative and HIV-positive African women (Adebamowo et al., 2017). Also, the Respiratory Microbiota of African Children (ReMAC) based at the University of Cape Town in SA.

The South African Council for Scientific and Industrial research (CSIR) in collaboration with the Sydney Brenner Institute for Molecular Bioscience at the University of the Witwatersrand have recently (2021) launched the CSIR Microbiome Mapping Initiative (CMMI) which aims to combine machine learning, 3rd generation sequencing technologies, environmental modelling and bioinformatics to better understand the relationships between gut health and over 100 lifestyle, health and environmental factors in South Africa by recruiting ∼500 participants based in the metros of Johannesburg and Pretoria (CMMI, 2021). It is our hope that the CMMI project will provide a framework for the coordination of larger national microbiome profiling efforts that could ultimately adopt a citizen science funding model akin to the AGP that would support its sustainability and also promote public awareness around microbiome research and its significance to the future of human health.

## Future Perspectives and Lessons From Our Past

Publication of the first complete human genome sequence (Venter et al., 2001) launched genomics into the 21st century, and with it the promise of genomic medicine. Precision medicine became a reality by extracting unique sequence-specific variants which deviated from the “baseline” of the initial sequences published—a baseline extracted from just five samples of European origin. However with accessibility to more diverse datasets, it became clear that the human genome harboured significant inter-individual and inter-ethnic diversity which would in turn have profound effects on disease risk and clinical outcomes. This is best exemplified by the initial 1,000 Genomes Project revealing that African genomes contain 25% more genetic variants than any other ethnic group (Gurdasani et al., 2015). Despite this revelation less than 2% of all currently analysed genomic datasets are of African origin (Sirugo et al., 2019). Thus, while precision medicine has been rapidly making improvements to the health space, much of the most advanced interventions are still based on well-established genomic profiles of Caucasian ancestry which do not adequately reflect global diversity, especially African diversity (Sirugo et al., 2019). The consequence of which is that under-researched genetic variants with higher prevalence in developing countries have significantly contributed to undesirable outcomes and perpetuated historical inequalities in healthcare across the African continent. With the microbiome positioned as the “second genome”, it also represents a second chance for African researchers to take stewardship of the continent’s healthcare narrative and prioritise the development of technologies and datasets that drive the African health agenda.

This will require a concerted investment from African researchers, funders and policy makers alike to avoid perpetuating the historical marginalisation of our continent and its unique healthcare needs within the evolving global healthcare arena. An important first step towards this goal would be the establishment of an African microbiome mapping initiative that embodies the “One Continent: One vision” ethos of this special issue. In contrast to the position the continent found itself in at the start of the genomic medicine revolution decades ago—improved access to NGS technologies and infrastructure along with established expertise in bioinformatics and microbiomics across numerous African research institutes—the continent is currently well poised to successfully meet this challenge. We invite all who share this vision to build the future of African precision medicine with us, pamoja!

## Data Availability

The original contributions presented in the study are included in the article/Supplementary Material, further inquiries can be directed to the corresponding author.
